# Ultra-Hypofractionated Prostate Radiotherapy With Online Adaptive Technique: A Case Report

**DOI:** 10.7759/cureus.64101

**Published:** 2024-07-08

**Authors:** SA Yoganathan, Mohamed Riyas, Renilmon Sukumaran, Rabih Hammoud, Noora Al-Hammadi

**Affiliations:** 1 Radiation Oncology, Hamad Medical Corporation, Doha, QAT

**Keywords:** cyberknife, ethos, adaptive, online, ultra-hypofractionated, prostate

## Abstract

Ultra-hypofractionated radiotherapy (UHF RT) is revolutionizing the treatment approach for low- and intermediate-risk prostate cancer patients. This study reports the planning process of UHF RT utilizing the cone beam computed tomography (CBCT)-based online adaptive radiotherapy (OART) treatment with the Ethos system, focusing on a comparative analysis between OART and image-guided radiotherapy (IGRT) plans. We also assessed the pre-planning capabilities of the Ethos system against the CyberKnife (CK) (Accuray, Sunnyvale, CA) system. A 66-year-old patient, diagnosed with prostatic acinar adenocarcinoma confirmed via biopsy and presenting with elevated prostate-specific antigen (PSA) levels, underwent UHF OART treatment using the Ethos system. The planning encompassed delineating the gross target volume (GTV) as the prostate, while the clinical target volume (CTV) comprised the prostate and proximal seminal vesicle. The planning target volume (PTV) was derived from the CTV with a 5 mm external margin except for a 3 mm posterior margin. A simultaneous integrated boost (SIB) technique was employed, delivering 40 Gy in five fractions (8 Gy per fraction) to the gross tumor volume (GTV) and 36.25 Gy in five fractions (7.25 Gy per fraction) to the remaining part of the planning target volume (PTV), with treatments scheduled biweekly. We compared OART and IGRT plans and conducted a comparative analysis between Ethos planning and the CK system for pre-planning assessment. When comparing Ethos planning and CK plans, Ethos demonstrated slightly better target coverage and organ-at-risk (OAR) sparing. However, CK plans showed superior containment of low-dose spillage, particularly at 50% and 25% iso-doses, due to non-coplanar beam arrangements. Our results demonstrated that OART plans yielded superior target coverage and improved OAR sparing compared to IGRT plans. Notably, the entire OART process, from planning to delivery, was accomplished within 27 minutes. The Ethos OART system's ability to adapt to daily anatomical changes, efficient workflow, and superior OAR-sparing capabilities make it a promising option for prostate cancer treatment using UHF RT.

## Introduction

Ultra-hypofractionated radiotherapy (UHF RT) is rapidly transforming the treatment landscape for low- and intermediate-risk prostate cancer patients [1]. This advancement would not be possible without technological breakthroughs that enable the delivery of high-dose conformal radiation in fewer fractions [2].

Historically, UHF RT for the prostate relied on high-dose-rate brachytherapy and stereotactic body radiotherapy (SBRT) [3]. SBRT utilizes image-guided radiation therapy (IGRT) delivered by either using specialized robotic systems such as CyberKnife (CK) (Accuray, Sunnyvale, CA) [3] or conventional linacs with C-arms [4].

The recent introduction of online adaptive radiotherapy (OART) has opened exciting possibilities for UHF RT, particularly in prostate cancer treatment. OART allows for a reduction in PTV margins, minimizing radiation exposure to healthy surrounding tissues such as the rectum and bladder [5], thereby lessening treatment-related side effects.

While limited studies have examined using OART with MRI guidance for UHF RT [6-8], our study explores a unique case. We report on a patient successfully treated with UHF RT using OART on the Ethos system and provide a comparative replanning of the case using the CyberKnife system.

This study reports the treatment processes involved in UHF RT using the Ethos OART system, specifically focusing on comparing OART and IGRT plans. We also evaluated the pre-planning capabilities of the Ethos system against those of the CK system, analyzing the dosimetric advantages and disadvantages of each. Additionally, we considered the logistical aspects of both systems. Our findings provide valuable insights into the potential of OART to improve UHF RT for prostate cancer.

## Case presentation

Clinical presentation

**Figure 1:**
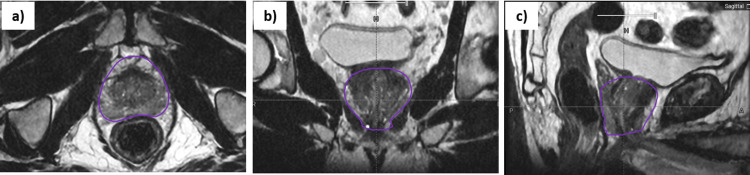
Magnetic resonance images show the enlarged prostate in axial (a), coronal (b), and sagittal (c) views.

A 66-year-old patient presented with high prostate-specific antigen (PSA) levels, and a biopsy confirmed prostatic acinar adenocarcinoma. The Gleason score was 3+4=7, 3/14 core positive, and prostate-specific antigen of 4.5 ng/mL with no evidence of lymph node involvement or distant metastases and WHO performance status of 0. Magnetic resonance imaging (MRI) showed an enlarged prostate with 35 cc in volume and multiple suspicious lesions in the peripheral and anterior parts of the prostate (Figure 1). Based on the decision of the multidisciplinary team meeting, he was planned to the UHF RT.

Pre-planning

The patient was prepared with a bladder protocol of 90 mL of water in 15 minutes. Although we aimed to get consistent bladder volume, we instructed the patient not to stress himself, as the OART would account for the change in bladder volume. The patient was supine with our institutional positioning devices, and a planning computed tomography (CT) of slice thickness 1 mm was acquired. Also, T2-weighted and diffusion-weighted MRI images were acquired for planning purposes. The anatomical area needed for full dose calculation was only included, and additional slices in cranio-caudal direction were excluded; this is to speed up the oncouch adaptation process.

All CT and MRI images were imported into the Eclipse treatment planning system (TPS) for contouring. MRI images were registered rigidly with CT images, which would be called during oncouch adaptation later. Tumors and organs at risk (OAR) were contoured by a qualified radiation oncologist (RO) and reviewed by an independent RO. The OARs included the bladder, rectum, bowel (including sigmoid), penile bulb, femoral heads, and nerve vascular bundle. In addition, the prostate urethra was also contoured, and a 2 mm external margin was given to derive the prostate urethra planning risk volume (PRV). The gross target volume (GTV) included the prostate. The clinical target volume (CTV) included the prostate and the proximal seminal vesicle.

The final contours, CT, and MRI images were imported into Ethos treatment management (TPS) for online adaptive pre-planning. In Ethos, the planning target volume (PTV) was defined from the CTV as a derived structure with an external margin of 5 mm all around, except 3 mm posterior direction. The derived structure would be generated daily with the same margin, which necessitates that accurate contouring is needed only for the CTV during oncouch adaptation. A simultaneous integrated boost (SIB) was planned, where the GTV received a dose of 40 Gy, delivered in five fractions (8 Gy per fraction), while the remaining part of the PTV received 36.25 Gy in five fractions (7.25 Gy per fraction), with treatments scheduled twice weekly.

The Ethos TPS needs a clinical goal to optimize the pre-plan and the daily online adaptive plans. Table 1 shows the clinical goals that we used for planning this case. After the clinical goal definition, pre-plan optimization was done in the "dose preview" available in Ethos. Ethos optimization uses an intelligent optimization engine combined with a photon optimizer and Fourier transform dose calculation to generate fast fluence-map optimized dose distribution for a nine-field fixed-field intensity-modulated radiotherapy (IMRT) plan geometry. Here, the order of clinical goals was optimized to obtain maximum plan quality. After the optimization, the clinical goal and their order were authorized, and the same optimization parameters were used during oncouch adaptation. Final plan generation was followed by the optimization, and plan dose distribution was calculated using the Acuros algorithm. Ethos offers three fixed-field IMRT plans (7, 9, and 12 fields) and two volumetric modulated arc therapy (VMAT). For this case, we choose a three-arc VMAT plan for treatment.

**Table 1:** Clinical goals used for planning. Priority 1 indicates the most important goals, Priority 2 indicates very important goals, and Priority 3 indicates important goals. GTV: gross tumor volume, PTV: planning target volume, PRV: planning risk volume, Dmax: dose maximum, D0.1cc: dose to 0.1 cc volume, D1cc: dose to 1 cc volume, Dmean: mean dose, V97%: volume receiving 97% of the prescribed dose, V95%: volume receiving 95% of the prescribed dose, V38Gy: volume receiving 38 Gy, V37Gy: volume receiving 37 Gy, V36Gy: volume receiving 36 Gy, V34.4Gy: volume receiving 34.4 Gy, V32.6Gy: volume receiving 32.6 Gy, V30Gy: volume receiving 30 Gy. V29Gy: volume receiving 29 Gy, V20Gy: volume receiving 20 Gy, V18Gy: volume receiving 18 Gy

Structure	Goal	Priority
GTV 40 Gy	Dmax <102%-108%	1
	V97% >97%-95%	
PTV 36.25 Gy	V95% >96%-95%	1
	D1cc <103%-110% (cropped from GTV+2 mm)	
Prostatic urethra PRV	Dmax <38-40 Gy	1
Bladder	Dmax <38-40 Gy	1
	V37Gy <10-20 cc	2
	V32.6 <7%-10%	2
	V18Gy <35%-40%	2
	Dmean <15-25 Gy	3
	D0.1cc <34.4-35.5 Gy	3
Rectum	Dmax <38-40 Gy	1
	V36Gy <1-2 cc	2
	V34.4Gy <2-3 cc	2
	V32.6 <7%-10%	2
	V29Gy <18%-20%	2
	V18Gy <45%-50%	2
	Dmean <15-30G y	3
	D0.1cc <34.4-35.5 Gy	3
Bowel	V30Gy <0.5-1 cc	2
	V18Gy <4-5 cc	3
Penile bulb	V29Gy <50%-75%	2
Femoral heads	V20Gy <8-10 cc	3
Nerve vascular bundle	V38Gy <50%-75%	2

Online adaptive treatment delivery

During every session, the patient followed the same bladder protocol, with bladder volume verified using a handheld ultrasound imaging system before the imaging. The patient was comfortably positioned on the Ethos treatment couch with appropriate positioning devices. Cone beam computed tomography (CBCT) images were acquired using the "pelvis large" protocol with "iterative reconstruction" enabled. After the image acquisition, the AlignRT InboreTM attached to the Ethos system monitored the patient's position in real time throughout the delivery.

The Ethos system automatically contoured the influencer structures: prostate, seminal vesicle, rectum, bladder, and bowel. These auto-contoured influencers were reviewed and potentially edited manually by the RO. Specifically, the Ethos system contours the full seminal vesicles by default, but the superior slices were excluded to define only the proximal seminal vesicles. Following the influencer process, the target review process began. In this case, the GTV was the prostate, which was automatically derived. The CTV combined the prostate and proximal seminal vesicles, also derived automatically. Similarly, the PTV was derived from the CTV using the same margin defined during the pre-planning phase.

During the target review process, the RO utilized the edit target option to examine the targets on MRI images, which had been fused with the planning CT during the pre-planning phase. They verified the contours of the prostate and prostate urethra, finding that no adjustments were needed across any of the sessions. Other OARs, including the neurovascular bundle, penile bulb, and femoral heads, were contoured using deformable image registration between the planning CT and CBCT. The planning CT was deformed to align with the CBCT, creating a synthetic CT that was used for online dose optimization and calculation.

Once the contours were generated, the Ethos system automatically initiated the creation of a new treatment plan. It used the same clinical goals and beam geometry (three-arc VMAT) established during the pre-planning process. The adaptive plan's dose distribution was calculated using the Acuros algorithm. Ethos generated two plans: a scheduled plan and an adaptive plan. The scheduled plan, known as the "IGRT" plan, recalculated the original pre-plan based on the current session's anatomy, while the adaptive plan was entirely re-optimized. The new adaptive plan required clinical approval from the RO and technical approval from a medical physicist. After approval, the adaptive plan was transferred to the Mobius3D system for secondary dose calculation verification. Mobius recalculated the dose distribution using the collapsed cone algorithm and compared the results with the original Ethos calculation, employing a 3%/3 mm gamma evaluation criteria.

Finally, another CBCT scan was acquired for final patient position verification before beam delivery, and couch shifts were applied if needed. In this case, the adaptive plans were consistently superior to the scheduled plans in all sessions; thus, adaptive plans were chosen for delivery. After each session, log files were collected for the delivered adaptive plan, and the delivered dose was recalculated using the post-log files and evaluated using Mobius3D with a 3%/3 mm gamma evaluation. It is important to note that the patient's position was continuously monitored using the AlignRT system throughout the entire adaptive process. This ensured that the patient remained still after the acquisition of the first CBCT.

CyberKnife planning

A comparison of Ethos planning with the CyberKnife (CK) (Accuray, Sunnyvale, CA) system was also conducted. It is important to note that this comparison pertains only to the pre-planning phase, as the CK system currently does not support adaptive planning. The same patient was replanned using the VOLO optimizer available in the Precision planning system (Accuray, Sunnyvale, CA) for delivery with the M6 CyberKnife machine equipped with the InCise 2 multileaf collimator (MLC) system. The planning process aimed to achieve similar dose constraints as specified in Table 1. The planning parameters for the CyberKnife system included 52 nodes, 52 beams, and 58 segments, with a minimum monitor unit (MU)/segment of 125.8 and a maximum MU/segment of 783. All final dose calculations were performed using the RayTracing dose calculation algorithm. This comparison highlights the capabilities and limitations of each system in meeting the treatment objectives.

## Discussion

**Figure 2:**
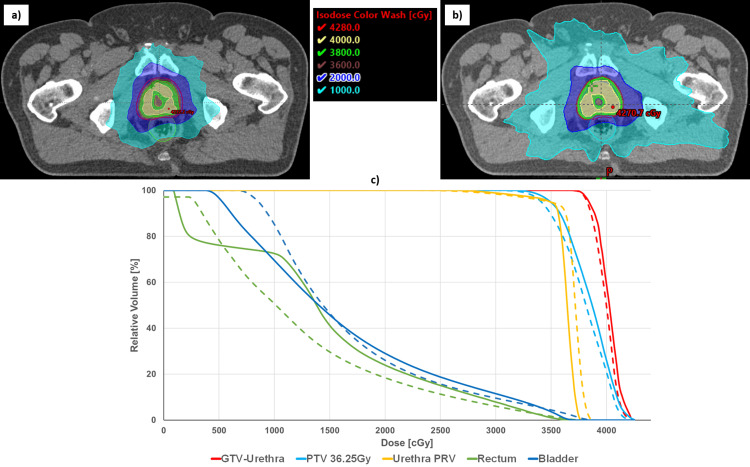
Dose distribution comparison on a CT image of the patient used in this study is shown between the CyberKnife plan (a) and the Ethos plan (b). This is accompanied by the cumulative DVH plot (c) of a session, where the solid line represents the Ethos plan and the dotted line represents the CyberKnife plan. DVH: dose-volume histogram, CT: computed tomography

Figure 2 illustrates the dose distribution and dose-volume histogram (DVH) comparison between the CK plan and the Ethos plan for pre-planning. Our findings indicate that the Ethos provided slightly superior dose coverage for the targets. The average GTV (excluding urethra PRV) coverage (V97%) was 86% in CK plans compared to 91.3% in Ethos plans. Similarly, the PTV coverage (V95%) was 93% in CK plans versus 97% in Ethos plans.

The CK system struggled with SIB due to limited dose modulation capabilities, making it inherently challenging to generate two dose-level treatments. In contrast, the Ethos system's multileaf collimator (MLC) facilitated effective dose modulation, allowing for SIB while sparing critical structures. The Ethos plans consistently resulted in lower maximum doses of OARs such as the bladder, rectum, and urethra. For CK plans, the Dmax values for the bladder, rectum, and urethra were 40 Gy, 39 Gy, and 39 Gy, respectively, while the corresponding values for Ethos plans were 37 Gy, 37 Gy, and 38 Gy, respectively.

The low-dose spill (50% and 25% iso-doses) was more favorable with CK plans due to the non-coplanar beam arrangements. Specifically, the rectum V18Gy was lower at 22.2% for CK, compared to 28.8% for Ethos. However, these non-coplanar beam arrangements in CK plans led to a higher bowel V5Gy, with CK at 58% versus 38% for Ethos. Additionally, the total monitor units (MUs) required were significantly higher for CK plans (15,253 MU for CK versus 2,639 MU for Ethos).

While the CK system delivered lesser low-dose spills through daily pre-planned treatments, Ethos offered superior OAR sparing with daily patient-specific optimization using OART. This is achieved through 3D anatomical imaging via CBCT and real-time AlignRT surface guidance, eliminating the need for invasive fiducial placement and extensive bladder and rectum preparation. Ethos further enhanced the precision with MRI integration for online adaptive sessions, allowing RO to ensure accurate contouring and thus increasing their confidence. Additionally, deformable image registration on the Ethos system enabled comprehensive evaluation of delivered dose accumulation over all fractions. These features collectively make Ethos superior in ensuring precise OAR sparing.

**Figure 3:**
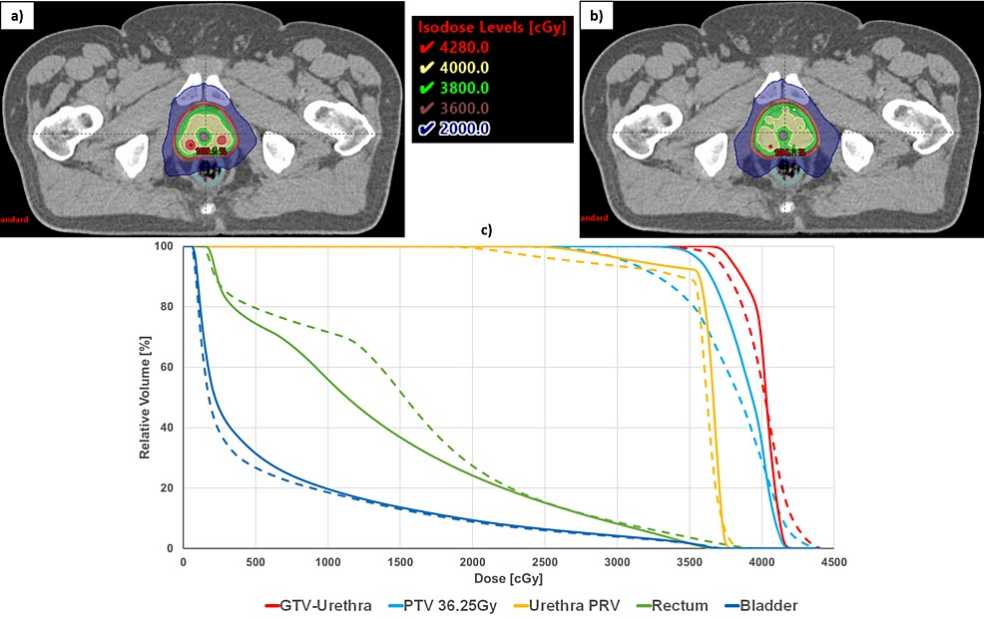
Dose distribution comparison on a CT image of the patient used in this study is shown between an IGRT plan (a) and adaptive plan (b), accompanied by the cumulative DVH plot (c) of a session. The solid line represents the adaptive, while the dotted line represents the IGRT plan. DVH: dose-volume histogram, CT: computed tomography, IGRT: image-guided radiotherapy

Next, we compared the treatment delivered to the patient, specifically examining the IGRT versus adaptive plans delivered using Ethos over five sessions. Figure 3 shows the dose distribution and DVH comparison for a session, indicating that the adaptive plan offered superior target coverage while effectively sparing critical organs. Table 2 presents a comparison of achieved clinical goals between IGRT and ART for a specific fraction. The average GTV-Urethra coverage (V97%) was 72±5% in IGRT plans compared to 85±4% in adaptive plans. Similarly, the PTV coverage (V95%) was 84±5% in IGRT plans versus 97±2% in adaptive plans.

**Table 2:** Comparison of achieved clinical goals between IGRT and adaptive plans for a specific fraction. IGRT: image-guided radiotherapy

Structure	Clinical goal	Tolerance	IGRT	ART
GTV 40 Gy	Dmax (Gy)	102%-108%	111	106
	V97% (%)	97%-95%	78	88
PTV 36.25 Gy	V95% (%)	96%-95%	84	98
	D1cc (Gy)	103%-110%	112	109
Prostatic urethra PRV	Dmax (Gy)	7.8-8 Gy	8.1	7.8
Bladder	Dmax (Gy)	7.6-8 Gy	8.1	7.7
	V7.4Gy (%)	10-20 cc	0.7	0.2
	V6.5Gy (%)	7%-10%	4.0	3.3
	V3.6Gy (%)	35%-40%	13.2	11.0
	D0.1cc (Gy)	34.4-35.5 Gy	7.6	7.4
Rectum	Dmax (Gy)	38-40 Gy	8.2	7.6
	V7.2Gy (cc)	1-2 cc	1.3	0.4
	V6.9Gy (cc)	2-3 cc	2.2	1.2
	V6.5Gy (%)	7%-10%	6.0	5.0
	V5.8Gy (%)	18%-20%	10.0	9.4
	V3.6Gy (%)	45%-50%	35.0	29.0
	D0.1cc (Gy)	34.4-35.5 Gy	7.9	7.3
Bowel	V6Gy (cc)	0.5-1 cc	0.0	0.0
	V3.6Gy (cc)	4-5 cc	0.0	0.0
Penile bulb	V5.8 (%)	50%-75%	0.0	0.8
Femoral heads (right)	V4Gy (cc)	8-10 cc	0.0	0.0
Femoral heads (left)			0.0	0.0
Nerve vascular bundle (right)	V7.6Gy (%)	50%-75%	0.4	3.9
Nerve vascular bundle (left)			0.0	9.6

The adaptive plans resulted in OAR doses (Dmax) that were 5% and 8% lower for the bladder and rectum, respectively, compared to IGRT plans. The doses received by the bowel, femoral head, and nerve vascular bundle were nearly identical in both plans, with differences of less than 1%. However, the penile bulb was better spared in the adaptive plans (V29.5Gy: 14.7% versus 4%).

The average MUs for adaptive plans were 2,669 (ranging from 2,265 to 2,926). The Mobius gamma evaluation pass rate exceeded 99% for all adaptive sessions. The average 3D couch shifts applied after the second verification CBCT imaging were less than 1 mm. Post-delivery log file analysis confirmed the accurate delivery of adaptive plans, with over 99% of pixels passing the gamma criteria for all sessions.

**Figure 4:**
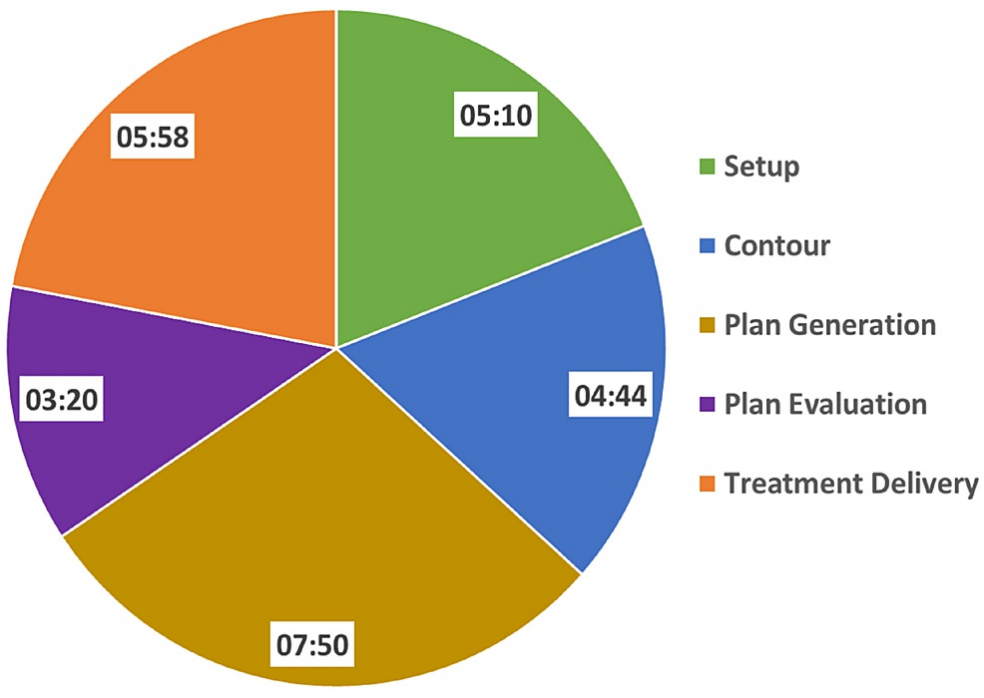
Ethos online adaptive workflow duration for an individual process, with all values presented as average times in minutes.

While MRI-guided OART approaches exist [6-8], our study highlights the efficiency of the Ethos CBCT-based OART system for UHF RT of the prostate. Although MRI may offer superior image quality, it requires slightly more time for OART, averaging 38.2 minutes (range: 33.3-43.4 minutes) for step-and-shoot intensity-modulated treatments [8]. In contrast, Ethos OART treatment sessions were completed within a tight timeframe (25-30 minutes) using VMAT, as shown in Figure 4. This compares favorably to other reported methods: Calvo-Ortega et al. [9] recently reported an online adaptive method for the treatment of UHF RT of the prostate using a conventional C-arm linear accelerator. Their findings demonstrated improved target coverage with adapted plans and an average treatment duration of 28±7 minutes. Similarly, the CK plan in our study required a comparable delivery time of approximately 26 minutes.

In our study, we opted for a three-arc VMAT plan for treatment due to our institution's expertise with VMAT for IGRT prostate treatments. Also, our initial comparisons of DVH parameters between VMAT and IMRT for OART showed similar outcomes, with VMAT notably reducing low-dose streaks present in the 12-field IMRT setup. While VMAT plan generation during adaptive sessions in Ethos typically took 9-10 minutes versus 2-3 minutes for IMRT, VMAT delivery time was faster (around nine minutes compared to 14 minutes for 12-field IMRT), resulting in similar overall treatment durations. A noteworthy challenge was the time-consuming contouring and planning process inherent to OART, leading to potential variations in bladder filling between initial and pre-treatment CBCT scans. Despite these variations, our clinical focus remained on achieving precise target alignment for optimal coverage of the prostate and proximal seminal vesicles. Importantly, bladder volume changes primarily occurred at the superior aspect, typically outside the treatment field, minimizing dosimetric impact.

## Conclusions

We reported our experience in treating prostate cancer using UHF RT with the Ethos OART system. The comparison of Ethos planning performance with CK plans revealed that Ethos provided slightly superior target coverage and sparing of OAR, while CK plans offered better low-dose spill. Furthermore, our findings demonstrated that OART plans yielded superior target coverage and improved OAR sparing compared to IGRT plans. Notably, the entire OART process, from planning to delivery, was efficiently completed within just 27 minutes. While our study supports the advantages of the Ethos OART system, future research on a larger population is necessary to confirm these results.
